# Role of Ondansetron in Obsessive-Compulsive Disorder: A Systematic Review

**DOI:** 10.1192/bjo.2025.10113

**Published:** 2025-06-20

**Authors:** Neelam Choudhary, Nilamadhab Kar

**Affiliations:** ^1^Black Country Healthcare NHS Foundation Trust, Dudley, United Kingdom; ^2^Black Country Healthcare NHS Foundation Trust, Wolverhampton, United Kingdom; ^3^University of Wolverhampton, Wolverhampton, United Kingdom

## Abstract

**Aims:** In obsessive-compulsive disorder (OCD), treatment non-response is common with recommended medications; hence there is a need to investigate the role of alternative agents. It was intended to evaluate the evidence base involving ondansetron as a treatment strategy for OCD in adults, considering its role as a 5-HT3 receptor antagonist primarily in the limbic system.

**Methods:** Various electronic databases (PubMed, CINAHL, EMBASE, PsycInfo, Cochrane, Clinical Trials Registers, and PROSPERO) were searched for articles with keywords of ‘ondansetron’, ‘obsessive-compulsive disorder’ or ‘OCD’, and ‘clinical trial’, in the English language, published up to October 2024.

**Results:** Nine studies were included in this systematic review. Studies used Yale–Brown Obsessive Compulsive Scale (YBOCS) scale for assessment and ondansetron mostly as an augmenting drug. More than half of the studies (five out of nine) had patients with treatment-resistant OCD, and serotonin reuptake inhibitors as the active drug. All trials reported improvement in OCD with ondansetron augmentation, except the study which involved chart review for treatment-resistant OCD. The improvement was observed as early as two weeks following ondansetron augmentation. The reported range of decrease in YBOCS score from baseline in various studies has been 23.2–55%. Treatment response was reported between 37–86.4%. Improvement was noticed in both OCD and treatment-resistant OCD groups. Discontinuation of ondansetron led to worsening of symptoms which was reported in two studies. Overall adverse effects of ondansetron were mild to moderate in degree, with no major clinical concerns, and were generally well tolerated. Commonly reported side effects were constipation, headache, diarrhoea, decreased appetite, dizziness, insomnia, anxiety, nervousness, sweating, dry mouth, muscle cramp and sexual dysfunction. Available studies have major limitations which are mostly small sample sizes and short duration of trials.

**Conclusion:** Current evidence suggests ondansetron may be beneficial in the treatment of OCD and treatment-resistant OCD, mostly as an augmentation agent; however the evidence is scarce to draw any firm conclusion. Further trials with adequate sample size and duration are required.

